# Effects of pregnancy on electrocardiographic, vasovagal tonus index, and echocardiographic variables in horses

**DOI:** 10.14202/vetworld.2023.1765-1771

**Published:** 2023-08-28

**Authors:** Chayanon Chompoosan, Pongphol Pongthaisong, Wootichai Kenchaiwong, Theerapong Pontaema, Wichaporn Lerdweeraphon

**Affiliations:** 1 Applied Animal Physiology Research Unit, Faculty of Veterinary Sciences, Mahasarakham University, Mahasarakham 44000, Thailand; 2 Small Ruminant Research Unit, Faculty of Veterinary Sciences, Mahasarakham University, Mahasarakham 44000,Thailand; 3 Network Center for Animal Breeding and Omics Research, Khon Kaen University, Khon Kaen 40002, Thailand

**Keywords:** echocardiography, electrocardiogram, horses, pregnancy, vasovagal tonus index

## Abstract

**Background and Aim:**

Pregnancy affects maternal hemodynamics. The changes in autonomic nervous system activity for hemodynamics adaptation in pregnant horses are still unclear. Thus, this study aimed to examine the effect of pregnancy on electrocardiographic, vasovagal tonus index, and echocardiographic variables in horses.

**Materials and Methods:**

A total of 23 Thai native crossbred mares without any cardiac abnormalities were included in this study. The animals were assigned into two groups, a non-pregnant mare group (n =12) and a pregnant mare group (n = 11). Electrocardiogram recordings (paper speed = 25 mm/s and calibration = 10 mm/mV) were performed to obtain six limb leads (leads I, II, III, aVR, aVL, and aVF). The vasovagal tonus index (VVTI) was calculated to assess variability in heart rate over short periods using just 20 consecutive beats. Cardiac structure and function were evaluated by echocardiography.

**Results:**

Heart rate, P wave duration, PR interval, QRS duration, QT interval, and T wave duration were significantly different between non-pregnant and pregnant horses (p < 0.05). Pregnant horses had significantly lower VVTI than non-pregnant (p < 0.05). There were no significant differences in cardiac structures including % interventricular septum (IVS), % left ventricular posterior wall (LVPW), IVS in diastole, left ventricular internal diameter at end-diastole, LVPW thickness at end-diastole, IVS in systole, left ventricular internal diameter at end-systole, LVPW thickness at end-systole, and left atrium/aortic roots ratio between the two groups. However, the pregnant horses had a significantly higher cardiac output and % ejection fraction than non-pregnant horses (p < 0.05).

**Conclusion:**

This study provided the first evidence that hemodynamic adaptations during pregnancy modified cardiac conduction, vasovagal tonus index, and echocardiographic variables in horses.

## Introduction

A cardiovascular function could be changed for physiological adaptation during pregnancy, including increases in heart rate (HR) [1], cardiac output (CO) [2], and a decrease in maternal systemic vascular resistance associated with the renin-angiotensin-aldosterone system activation [3]. The changes in cardiovascular function associated with pregnancy are relevant to clinical practice because they may be exacerbated in association with cardiovascular diseases, including hypertension [4, 5], diabetes mellitus [6], and congenital heart disease [7] and also lead to the development of pre-eclampsia in women [8–10]. Physiological heart murmurs commonly develop during pregnancy in dogs [11]. Studies of pregnant dogs have demonstrated that hemodynamic adaptations to pregnancy affect maternal electrocardiographic parameters [12] and echocardiographic variables, particularly an increase in HR and CO [11, 13–15]. This indicates that the main mechanisms of cardiac adaptation in pregnancy is germane to the activity of autonomic nervous system (ANS) [3] and is associated with a decrease in parasympathetic activity and an increase in sympathetic activity for the increase in blood volume to supply optimal uteroplacental blood flow [9]. However, how pregnancy affects maternal hemodynamics associated with changes in cardiac conduction is still unclear in horses.

The balance of sympathetic and parasympathetic activity can be analyzed by variability in heart rate or HRV for assessing stress and different welfare states in farm animals [16]. In addition, HR variability (HRV) is useful for diagnosing arrhythmias in sports horses [17, 18] and was widely used in horses [19–22]. Vasovagal tonus index or VVTI (time domain analyses of HRV) is a simple method of assessing sympathetic and parasympathetic tone balance in horses [23]. In a recent study in horses, maternal HRV did not change across the three trimesters but increased only after foaling [24]. However, the changes of HRV in normal horses and pregnant horses have not been examined and compared. Thus, this study was established to examine factors in time domain analysis of HRV.

The previous studies have demonstrated that hemodynamic adaptations to pregnancy affect maternal echocardiographic variables, increase CO [11], increase left ventricular wall thickness and left ventricular wall mass [25, 26], and increase left ventricular end-diastolic volume and left atrial area throughout pregnancy in women [27]. The left ventricular free wall in systole increased in normal bitches [14] and the left ventricular diastolic function changed throughout pregnancy in dogs [15]. These results indicate that cardiac remodeling can be associated with volume overload and, eventually, ventricular hypertrophy during pregnancy [28, 29]. However, little is known about the effect of pregnancy on cardiovascular function in horses. In addition, echocardiographic variables of healthy pregnant horses have not been investigated.

Therefore, this study aimed to examine the effect of pregnancy on electrocardiographic parameters, vasovagal tonus index, and echocardiographic variables in horses.

## Materials and Methods

### Ethical approval

The study was approved by Institutional Animal Ethics Committee, Mahasarakham University, Thailand (Approval number: IACUC-MSU-7/2023).

### Study period and location

The study was conducted from November to December 2022 at Husbandry Section of the 2^nd^ Livestock and Agriculture Division, Veterinary and Remount Department, Tha Phra Subdistrict, Mueang District, Khon Kaen Province, Thailand.

### Animals

A total of 23 Thai native crossbred mares aged 6.5 ± 2.6 years with body weight of 433.1 ± 39.0 kg were used in this study. The gestational stage of animals was evaluated by breeding history and transrectal ultrasound examination. Animals were assigned to two groups: a non-pregnant mare group (n = 12) and a pregnant mare group (n = 11). The pregnant mares were at gestational stages of 150–270 days (mean 235 ± 35.8 days). The exclusion criteria were any cardiac problems such as heart murmur, cardiac arrhythmias, and structural heart abnormalities (based on a result of auscultation, resting electrocardiogram (ECG) recording, and echocardiography).

### Electrocardiogram examination

Electrocardiogram recordings were performed on animals using a 3-channel electrocardiograph (Edan Instruments, Inc., VE-300, China) at a paper speed of 25 mm/s and calibration of 10 mm equal to 1 mV. Before recording, the animals were restrained using a halter in the station in a standing position without any chemical restraint allowing acclimation within 5 min. All horses had ECG recordings in the base-apex lead systems. Four electrodes were placed on unshaved skin with alligator clips for all the standard bipolar limb leads (leads I, II, and III) and unipolar augmented limb leads (lead aVR, aVL, and aVF). For the forelimb, The right atrium (RA) (white) electrode was positioned on the right jugular furrow or in front of right scapula spine and the left atrium (LA) (black) electrode was positioned on 5^th^ intercostal space, just behind the point of the elbow of the left forelimb. For the hindlimb, the LF (red) electrode was positioned on the loose skin at the left stifle in the region of the patella and the RF (green or grounding) electrode was placed on the skin at the xiphoid as described previously [30]. Alcohol was applied to improve electrical contact. The ECG was recorded for approximately 1 min for each lead system. The recording from lead I was used to evaluate HR and ECG parameter measurements, including P wave duration and amplitude, PR interval, QRS duration and amplitude, and QT interval. T wave morphology was observed with three patterns, which were positive and negative deflection and biphasic. Heart rate was evaluated using R-R interval. The same researcher performed the analysis of ECG recording.

### Calculation of HRV

This study used the vasovagal tonus index (VVTI) as a time domain indicator of HRV analysis. VVTI measurement used short-term recordings and may be appropriate for the resting horse [23]. The good quality ECG trace and continuous running of sinus rhythm at the first 20 consecutive R-R intervals of 1 min in each ECG recording were selected to measure HRV [31]. Heart rate variability or VVTI was obtained by calculating from the variance standard deviation of the R-R interval_2_, for this interval in milliseconds and the natural logarithm of the variance of the 20 measured R-R intervals [32], as described by the equation:

VVTI = NL [VAR (R-R_1_ - R-R_20_)], where NL is natural logarithm and VAR is variance.

### Cardiac function evaluations

Echocardiography was performed on an unsedated horse in a standing position using a portable ultrasound system (VINNO V6, China) with a phase array 1.0–4.0 MHz transducer. Two-dimensional images included the right parasternal long-axis 4-chamber view. M-mode images were obtained of the right parasternal short-axis view of the left ventricle (LV) at the chordal level as described in a previous study [33]. Echocardiographic images were captured and stored for offline analysis. All images were analyzed by a single observer. The mean of three cardiac cycles was calculated for all measurements and was used for further analysis.

A conventional echocardiographic protocol was performed following previously published guidelines [34]. The protocol included transthoracic two-dimensional, M-mode, and color-flow Doppler echocardiography without ECG. Color-flow Doppler echocardiography was used to evaluate the mitral, tricuspid, aortic, and pulmonic valves and detect regurgitation jets. The diameter of the LA and the diameter of the aortic roots (AO) were measured from the right parasternal short-axis view at the level of the aortic valve. The LA diameter was indexed to the AO diameter. Ventricular wall thickness and dimensions were measured during diastole and systole using M-mode obtained from the right parasternal short-axis view at the level of the papillary muscles [34]. These parameters included left ventricular internal diameter at end-diastole (LVIDd), left ventricular internal diameter at end-systole (LVIDs), left ventricular posterior wall thickness at end-diastole (LVPWd), left ventricular posterior wall thickness at end-systole (LVPWs), interventricular septum in diastole (IVSd), interventricular septum in systole (IVSs), and fractional shortening (FS%). The FS% was calculated using the formula (LVIDd-LVIDs) * 100/LVIDd. The left ventricular volume at end-diastole (EDV) and at end-systole (ESV) was calculated using Simpson’s method of disks from the right parasternal long-axis 4-chamber view [35]. The stroke volume (SV), LV mass (LVM), % interventricular septum (IVS), and % left ventricular posterior wall thickness (LVPW) were automatically calculated by the software. Stroke volume was calculated using the following formulas: SV = EDV–ESV and ejection fraction (EF%) was calculated using the following formula: EF% = (EDV–ESV) *100/EDV. The LVM was calculated using the following formula: LVM = 1.04*(LVIDd + LVFWd + IVSd)^3^ – LVIDd^3^) – 13.6 [36]. The %IVS was calculated using the following formula: %IVS = (IVSs – IVSd)/IVSd × 100. The %LVPW was calculated using the following formula: %LVPW = (LVPWs – LVPWd)/LVPWd × 100. The CO was calculated using the following formula: SV* HR (HR was evaluated using R-R interval from ECG recording).

### Statistical analysis

The normality of all parameters in our study was tested by QQ plot normal and Shapiro-Wilk.

Data analysis was performed under a one-way analysis of variance experimental plan using the generalized linear models with SAS University Edition (SAS/STAT^®^, SAS Institute Inc., NC, USA). The amount of ECG data from the two groups of horses is unequal, which is an unbalanced design. Therefore, the analysis of the experimental data to summarize the effect of the factors and the linear contrast test with the least square mean (LS-means) are described. The LS-means between the two independent groups were compared by the least significant difference method. Descriptive statistics of vasovagal tonus index utilized a Kruskal-Wallis test. p < 0.05 was considered statistically significant. The coefficient of variation is the ratio of the standard deviation to the mean. The effects of treatments (pregnant and non-pregnant) were analyzed in relationship with ECG and echocardiographic observations. In the analytical model, covariate variables of weight and age were analyzed with adjustments to test the effect of treatments using the following equation:


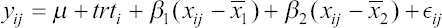


Where,*y_ij_* is the observation of the treatment *i*^th^, replicated *j*^th^; *µ* is overall mean, *β*_1_, *β*_2_ is regression coefficient of age and body weight; *trt* is effect of treatment (pregnant-not pregnant) *x_ij_* is covariate of treatment treatment *i*^th^, replicated *j*^th^; *x̅*_1_, *x̅*_2_ is mean of age and weight and ϵ*_ij_* is random errors.

## Results

Testing the relationship between age factor and body weight on ECG and echocardiographic parameters, this study found only age factor significantly related with QRS duration of ECG parameters (p = 0.03). All ECG parameters were expressed as least square mean ± standard error (Table-1). There were significant differences between the non-pregnant and pregnant horses in HR, P wave duration, PR interval, QRS duration, QT interval, and T wave duration (p < 0.05). There was an increase in HR in pregnant horses compared to the non-pregnant horses. The pregnant horses had a lower duration of P, QRS, and T waves and the interval of PR and QT than the non-pregnant horses. T wave morphology was almost expressed in a biphasic configuration in both the non-pregnant and pregnant horses.

**Table-1:** The electrocardiographic parameters (least square mean ± standard error) of the non-pregnant and pregnant horses.

ECG parameters	Non-pregnant (n = 12)	Pregnant (n = 11)	p-value
HR (beats/min)	36.19 ± 2.35	53.5 ± 2.47	0.0001
P wave duration (s)	0.15 ± 0.007	0.12 ± 0.007	0.023
P wave amplitude (mV)	0.24 ± 0.020	0.22 ± 0.021	0.66
PR interval (s)	0.31 ± 0.012	0.25 ± 0.013	0.005
QRS duration (s)	0.13 ± 0.005	0.09 ± 0.005	0.0002
ORS amplitude (mV)	1.45 ± 0.081	1.60 ± 0.085	0.24
QT interval (s)	0.52 ± 0.012	0.43 ± 0.013	0.0003
T wave duration (s)	0.17 ± 0.008	0.11 ± 0.008	<0.0001

ECG=Electrocardiogram

**Figure-1:**
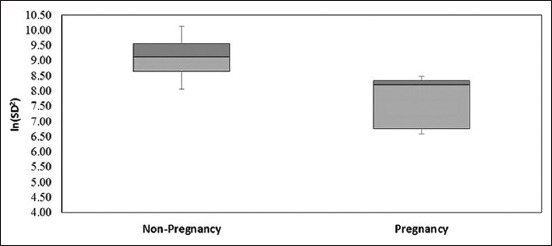
Box plot depicting the medians, interquartile ranges, and amplitude of vasovagal tonus index in non-pregnant and pregnant horses.

The comparison of VVTI values in non-pregnant and pregnant horses revealed a significant difference between the two groups. VVTI values of pregnant horses were significantly lower than non-pregnant (p < 0.05), as shown in Table-2 and Figure-1.

**Table-2:** Descriptive statistics of vasovagal tonus index in non-pregnant and pregnant horses.

Descriptive statistics	Non-pregnant	Pregnant	P-value (Kruskal-Wallis Test)
Minimum	7.8	6.6	-
25% percentile	8.7	6.8	-
Median	9.14 (9.74–8.49)	8.21 (8.45–6.75)	0.0038
75% percentile	9.6	8.4	-
Maximum	11.8	8.6	-
Coefficient of variation	11.40%	10.40%	-

**Figure-2:**
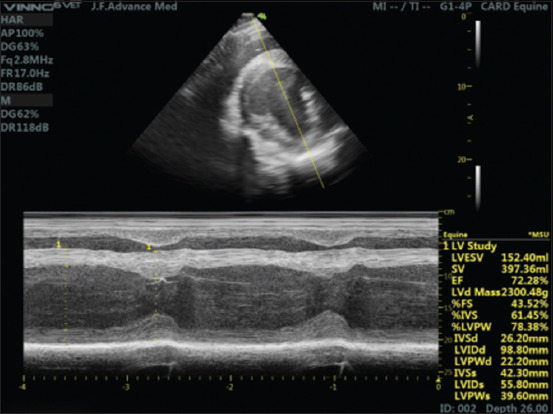
The right parasternal short-axis view of the heart in horses, during diastole, and during systole using M-mode echocardiography. IVS=Interventricular septum, LVID=Left ventricular internal diameter, LVPW=Left ventricular posterior wall.

Echocardiographic measurements from the right parasternal short-axis LV using M-mode echocardiography are shown in Figure-2. There was a significant increase in CO and EF in pregnant horses when compared to non-pregnant horses (p < 0.05). However, cardiac dimensions including %IVS, %LVPW, IVSd, LVIDd, LVPWd, IVSs, LVID at end-systole, LVPWs, and LA/Ao ratio were not different between the two groups (Table-3).

**Table-3:** Echocardiographic parameters (least square mean ± standard error) of non-pregnant and pregnant horses.

Echocardiographic parameters	Non-pregnant (n = 12)	Pregnant (n = 11)	p-value
SV (mL)	616.17 ± 33.42	650.49 ± 33.42	0.493
CO (L/min)	22.11 ± 1.41	35.31 ± 1.48	<0.0001
EF (%)	72.84 ± 1.17	79.90 ± 1.17	0.0005
FS (%)	41.81 ± 1.62	45.30 ± 1.62	0.1597
LVd Mass (g)	2535.62 ± 165.55	2504.35 ± 165.55	0.8991
IVS (%)	57.39 ± 6.69	66.76 ± 6.69	0.3519
LVPW (%)	49.69 ± 4.64	50.17 ± 4.64	0.9445
IVSd (mm)	24.85 ± 1.04	25.70 ± 1.04	0.583
LVIDd (mm)	98.59 ± 1.53	96.38 ± 1.53	0.3374
LVPWd (mm)	26.56 ± 1.32	26.84 ± 1.32	0.8879
IVSs (mm)	38.62 ± 1.60	42.50 ± 1.60	0.1138
LVIDs (mm)	57.33 ± 1.73	52.69 ± 1.73	0.084
LVPWs (mm)	38.90 ± 1.33	39.83 ± 1.33	0.6372
LA/Ao ratio	1.21 ± 0.05	1.16 ± 0.05	0.5482

LVIDd=Left ventricular internal diameter at end-diastole, LVIDs=Left ventricular internal diameter at end-systole, LVPWd=Left ventricular posterior wall thickness at end-diastole, LVPWs=Left ventricular posterior wall thickness at end-systole, EDV=Left ventricular volume at end-diastole, ESV=Left ventricular volume at end-systole, SV=Stroke volume, EF=Ejection fraction, FS=Fractional shortening, LA=Left atrium, IVSd=Interventricular septum in diastole, IVSs=Interventricular septum in systole, CO=Cardiac output

## Discussion

This study was conducted in the third or final trimester of pregnancy (average of 235 ± 35.8 days). This trimester is the most important trimester in terms of fetal growth and development might be significantly different when comparing hemodynamic adaptation between normal horses and pregnant horses. The main finding of this study was that there was a higher HR in pregnant horses while the duration of ECG waveform and interval of PR and QT in the pregnant horses were lower than those in the non-pregnant horses. Moreover, vasovagal tonus index (the time domain of variability in HR) was decreased during pregnancy in horses. Echocardiographic parameters were significantly different in CO and EF. However, cardiac morphological variables were not statistically significantly different between pregnant and non-pregnant horses.

In this study, the HR increased obviously during the late pregnancy in horses, similar to earlier reports in pregnant Warmblood mares [37], humans [1, 3, 38, 39] and in dogs [12, 15, 40]. In horses, the breed differences may reflect in maternal HR; the small-size mares had the highest HR compared to the medium-sized and large-sized mares [24]. This study found that the increase in HR values (pregnant = 53.5 ± 2.47 bpm and non-pregnant = 36.19 ± 2.35 bpm) of pregnancy in medium-sized Thai native crossbred mares was similar to a previous report [24]. Reference values of ECG parameters for normal Thai native crossbred horses in this study agreed with earlier reports [41]. We found that the duration of P, QRS and T wave and the interval of PR and QT were deceased in pregnant horses compared to non-pregnant horses. Changed electrical properties of the myocardium may be due to changes in the sympathetic activity, sex hormones (estrogen, progesterone) and ion channel expression, consequently affect ECG during pregnancy [42]. A previous study reported that PR interval decreased in the third trimester. The shortening of PR indicates an acceleration of atrioventricular node conduction velocity due to increased sympathetic activity resulting in tachycardia [43]. Sex hormone such as estrogen can alter the electrophysiological properties of the myocardium and changes in ventricular repolarization result in lengthening of the QT interval during pregnancy [42]. However, this alteration may not be explained shortening of QT in pregnant horses in this study. In addition, the changing cardiac rhythm may affect the ECG wave duration and interval, including PR and QT interval; there was a negative correlation between HR and PR intervals in dogs [44]. Similar changes have been found in HR, PR interval, and the QT interval showed significant differences between the untrained and trained horses [41, 45]. This implies that cardiac adaptation may alter the duration and interval of ECG wave, especially HR in pregnant mares. The main mechanism of cardiac adaptation in pregnancy involves the activity of ANS [3], which is associated with a decrease in parasympathetic activity and an increase in sympathetic activity, leading to an increase in blood volume to supply optimal uteroplacental blood flow [9]. The balance of sympathetic and parasympathetic activity can be analyzed by variability in HR for assessing stress and different welfare states in farm animals [16]. In addition, HRV has been reported as useful for diagnosing arrhythmias in horses [17, 18].

Vasovagal tonus index or VVTI (time domain analyses of HRV) is a simple method of assessing the balance of sympathetic and parasympathetic tone in horses [23] and thus was evaluated in this study. In a recent study, during pregnancy in horses, it was reported that the maternal HRV did not change across the first, second, and third trimester. However, the maternal HRV increased only after foaling, and the changes in HRV between normal horses and pregnant horses have not been examined [24]. Thus, this study was established to examine them and found that pregnant horses had significantly decreased VVTI (8.21, 8.45–6.75) compared to non-pregnant horses (9.14, 9.74–8.49). Decreased HRV is associated with reduced parasympathetic tone and increased sympathetic tone and may be considered a normal physiological response to pregnancy in this study as well as stress and exercise or excitement. In horses, VVTI is reduced (7.90 ± 0.9; n = 17) in valvular heart disease compared to a group of horses without cardiac disease (9.10 ± 0.9; n = 21) [46].

Hemodynamic adaptation during pregnancy can affect echocardiographic variables in humans [3] and dogs [13, 15, 47–49]. However, these effects have not been investigated during pregnancy in horses. This study found that echocardiographic variables changed in CO and %EF. However, SV, %FS, and the cardiac dimension variables did not change between pregnant and non-pregnant horses. There were significant increases in CO and %EF of pregnant horses compared to non-pregnant horses. The physiologic adaptation of increased CO may be due to increased total body water and blood volume during pregnancy [50]. Subsequently, a rise in HR maintains the increase in CO [51], even though SV did not significantly increase in pregnant horses in this study. We found that was a statistically significant increase in %EF but not %FS in pregnant horses, possibly reflecting the increased contractility of the heart to pump oxygen-rich blood volume from the left ventricle to support systemic organs, increased volume in each heartbeat, indicating the increase oxygen demand of uteroplacental circulation for fetal growth and development. This change in contractility of ventricle was altered by changes in ANS activity [52]. In Thoroughbreds, the increase in EF% was correlated with maximal oxygen uptake and LVM in racing horses [53, 54]. In this study, cardiac remodeling did not occur in pregnant horses. This is contradictory to previous studies in humans [25] and dogs [14]. There may be a species-dependent variability in maternal hemodynamic response. The horse has a higher vagal tone than other animal species such as dogs, rabbit, and mice, or humans [55], which might influence the ANS activity during pregnancy. However, this study did not examine the change in HRV and echocardiographic variables across trimesters of pregnant horses. Therefore, further study should aspire to examine the influence of states of pregnancy by considering sex hormones and serum electrolyte changes on electrocardiography, HRV and echocardiography in different breeds of horses. The limitations of this study were considered as the small number of horses sampled and hematological and urinary analysis for evaluating general health conditions. We have limited availability of equipment for simultaneous ECG during echocardiography. The echocardiographic technique was performed by a single observer and as there was no test for differences that might be obtained between different examiners, it is possible that repeatability was poor.

## Conclusion

This study established that hemodynamic adaptation during pregnancy can alter electrocardiographic variables, vasovagal tonus index or VVTI (balance of sympathetic and parasympathetic activity), and echocardiography in horses.

## Authors’ Contributions

WK: Designed the study and analyzed the data. CC, PP, TP, and WL: Recorded and analyzed the data. WL: Coordinated the study, wrote, and revised the manuscript. All authors have read, reviewed, and approved the final manuscript.
